# The Impact of Versatile Macrophage Functions on Acute Kidney Injury and Its Outcomes

**DOI:** 10.3389/fphys.2019.01016

**Published:** 2019-08-06

**Authors:** Jea-Hyun Baek

**Affiliations:** Research & Early Development, Biogen Inc., Cambridge, MA, United States

**Keywords:** macrophage, acute kidney damage, fibrosis, macrophage depletion, chronic kidney disease

## Abstract

Acute kidney injury (AKI) is a common and devastating clinical condition with a high morbidity and mortality rate and is associated with a rapid decline of kidney function mostly resulting from the injury of proximal tubules. AKI is typically accompanied by inflammation and immune activation and involves macrophages (Mϕ) from the beginning: The inflamed kidney recruits “classically” activated (M1) Mϕ, which are initially poised to destroy potential pathogens, exacerbating inflammation. Of note, they soon turn into “alternatively” activated (M2) Mϕ and promote immunosuppression and tissue regeneration. Based on their roles in kidney recovery, there is a growing interest to use M2 Mϕ and Mϕ-modulating agents therapeutically against AKI. However, it is pertinent to note that the clinical translation of Mϕ-based therapies needs to be critically reviewed and questioned since Mϕ are functionally plastic with versatile roles in AKI and some Mϕ functions are detrimental to the kidney during AKI. In this review, we discuss the current state of knowledge on the biology of different Mϕ subtypes during AKI and, especially, on their role in AKI and assess the impact of versatile Mϕ functions on AKI based on the findings from translational AKI studies.

## Introduction

**FIGURE 1:**
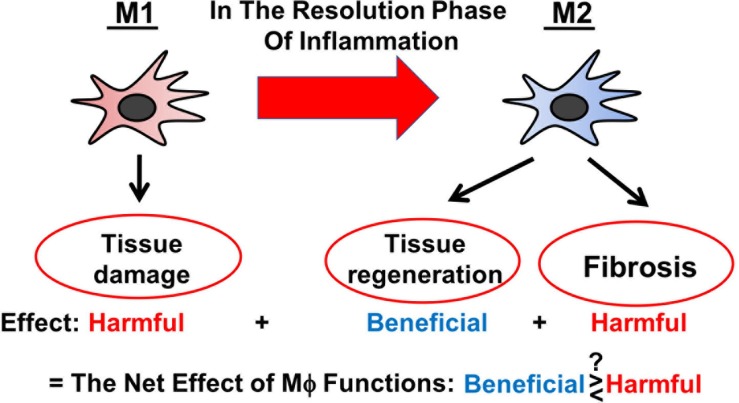
Schematic representation of versatile functions of Mϕ during AKI.

Severe AKI is a clinical condition closely linked with a high morbidity and mortality rate (Zuk and Bonventre, 2016). AKI manifests as a rapid decline of kidney function and is associated with CKD (Chawla et al., 2014; Fiorentino et al., 2018). AKI mostly results from the injury of proximal tubules and is accompanied by inflammation and immune activation (Zuk and Bonventre, 2019). Thereby, distinct Mϕ subtypes are involved across different stages of AKI (Huen and Cantley, 2017; Chen et al., 2019): (1) “Classically” activated (M1) Mϕ, which are poised to destroy potential pathogens, are recruited to the inflamed tissue and exacerbate inflammation in the initial stage of AKI; (2) “alternatively” activated (M2) Mϕ predominate in the injured tissue during the resolution phase of AKI and mediate immunosuppression and tissue regeneration; and (3) the last-mentioned also play a role in the transition of AKI to CKD. As M2 Mϕ are found to be protective against AKI, there is a growing interest to use M2 Mϕ and Mϕ-modulating agents as therapeutic tools to treat patients with AKI (Chen et al., 2019). Whilst valuing its immense therapeutic potential, it is to acknowledge that the clinical translation of Mϕ-based therapies needs to be critically reviewed and questioned, especially since Mϕ act like double-edged swords being both beneficial and harmful to the injured tissue (Figure 1) (Braga et al., 2015). In this review, we discuss the current state of knowledge on the biology of different Mϕ subtypes during AKI and on the impact of global Mϕ and Mϕ subtypes on AKI based on the findings from *in vivo* Mϕ depletion studies. At the end, we outline Mϕ-based therapeutic strategies for the treatment of AKI.

## Kidney Mϕ in Steady State and Inflammation

Mϕ are, as their name implies: [Greek: macrophage = *μακρ*ó*ς* (large) + *φαγε*í*ν* (to eat)], cells highly specialized in phagocytosis, belonging to the mononuclear phagocytic system (MPS). They reside in virtually all organs and orchestrate tissue homeostasis and inflammation, being capable of both inducing and suppressing immune responses as well as promoting tissue repair. Mϕ are also the most abundant leukocytes in the resting and inflamed kidney, maintained by two main Mϕ survival factors, CSF-1 and interleukin-34 (IL-34), primarily expressed by tubular epithelial cells (Isbel et al., 2001; Menke et al., 2009; Zhang et al., 2012; Baek et al., 2015). Both cytokines are further up-regulated during renal inflammation and account for Mϕ expansion of in the kidney tissue (Baek et al., 2015). CSF-1 and IL-34 both signal through the CSF-1 receptor (CSF-1R), whereas the signaling via CSF-1R is the key pathway for Mϕ proliferation, differentiation, and survival (Baek et al., 2015). In addition to CSF-1R, IL-34 activates another receptor, which is receptor-type tyrosine-protein phosphatase zeta (PTP-ζ), (Nandi et al., 2013). However, there is so far no evidence that PTP-ζ is expressed by Mϕ (Baek et al., 2015).

**FIGURE 2:**
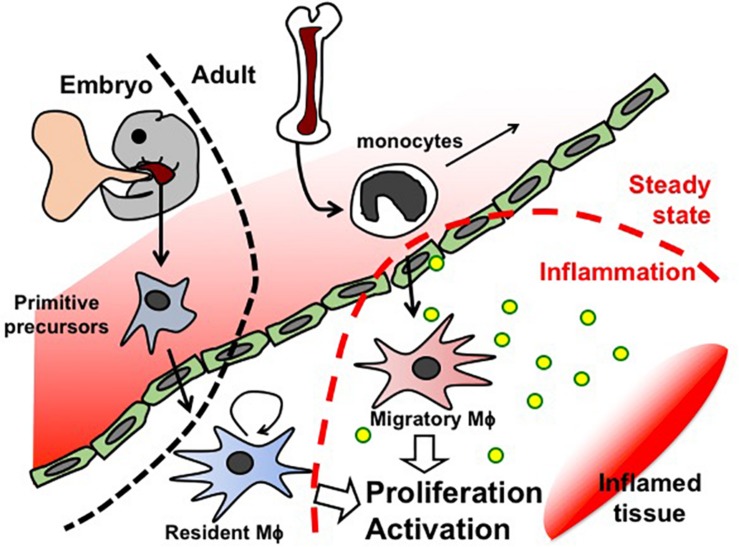
Schematic representation of Mϕ fate in embryogenesis and adults.

Like Mϕ in other organs, first kidney Mϕ arise during organogenesis, derived from erythro-myeloid progenitors that are generated in the yolk sac before E8.5 and colonize the fetal liver of the embryo. These primitive progenitors give rise to pre-Mϕ, which simultaneously populate the whole embryo from E9.5 and differentiate to fetal and perinatal tissue-specific Mϕ activating tissue-dependent transcriptional machinery (Mass et al., 2016). Tissue-resident Mϕ are known to renew themselves *in situ* throughout the lifetime of the host (Figure 2). However, Mϕ arising from blood-circulating monocytes (also known as circulating Mϕ precursors) are also detected in resting adult kidneys, but they turn over within 14 days and do not substitute kidney-resident Mϕ unless kidney-resident Mϕ niches become available (Lever et al., 2019).

In the occurrence of inflammation, additional Mϕ are recruited from the blood circulation (Sere et al., 2012). Blood-circulating monocytes are attracted to the site of inflammation, where they differentiate to Mϕ and clear pathogens and cellular debris (Figure 2).

**FIGURE 3:**
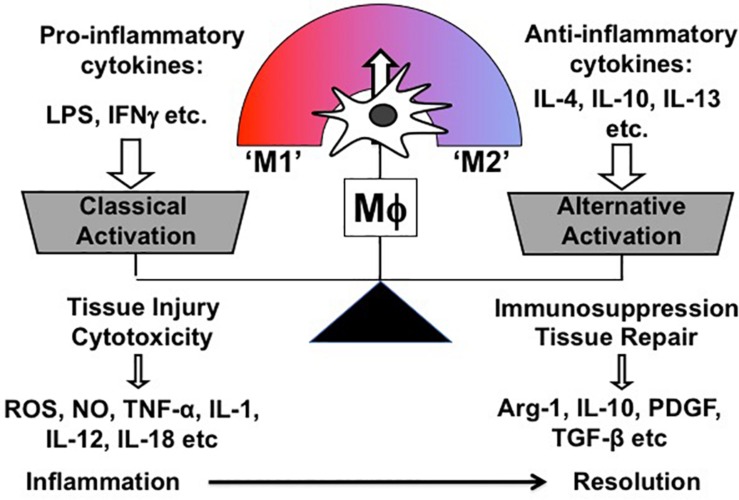
Schematic representation of Mϕ polarization *in vitro*.

Mϕ exert multiple biological functions in health and disease. Most importantly, they are instrumental in both promoting and resolving inflammation, which are two contrasting features. Correspondingly, Mϕ are broadly classified into two subpopulations according to their phenotype and function: So-called “classically activated” Mϕ (M1) are the Mϕ subpopulation inducing cytotoxicity and tissue injury; conversely, “alternatively” activated (M2) Mϕ comprise the other subpopulation, which is involved in immunosuppression and tissue repair (Mills et al., 2000; Murray and Wynn, 2011). Overall, M1/M2 paradigm is a theoretical and oversimplified concept, which was firstly proposed by Mills et al. based on the observation that Mϕ from mouse strains with Th 1 (e.g., C57BL/6, B10D2) and Th2 (e.g., BALB/c, DBA/2) display distinctive activation profiles differing in metabolic programs (Mills et al., 2000). Correspondingly, both Mϕ phenotypes were named M1 and M2 and characterized *in vitro* by stimulating bone marrow or monocyte-derived Mϕ with either Th1 (e.g., LPS, Interferon γ) or Th2 stimuli (e.g., IL-4, IL-10, IL-13) (Figure 3). In addition, M2-activated Mϕ are further subdivided into different groups based on the Th2 stimulus, with which Mϕ are treated for M2 polarization. Of note, different M2 stimuli have distinct effects on transcriptional profiles and cellular functions of Mϕ (detailed information in Murray et al., 2014; Chen et al., 2019).

One of the unique characteristics of Mϕ is functional plasticity. In other words, Mϕ can easily change their phenotype from one activation state to the other. Thus, *in vivo* Mϕ are, in reality, somewhere along the continuum between the two *in vitro*-defined phenotypes (M1 and M2), and *in vitro* polarized Mϕ do not fully recapitulate *in vivo* Mϕ in pathologic conditions (Figure 3) (Geissmann et al., 2010).

As Mϕ play important roles in many biological processes, their malfunction is linked to various diseases. While Mϕ-mediated immune hyper-activation can lead to autoimmune and inflammatory diseases, unregulated tissue homeostasis can promote cancer growth and organ fibrosis. Accordingly, Mϕ are implicated in numerous renal diseases including lupus nephritis, glomerulonephritis as well as in AKI (Nikolic-Paterson and Atkins, 2001; Baek et al., 2018).

## Experimental AKI Models

AKI is characterized by an abrupt loss of kidney function arising from different events, such as (1) sepsis/septic shock, (2) ureteral obstruction, (3) kidney ischemia, (4) hypoxia, (5) nephrotoxicity, (6) oxidative, and (7) metabolic stress. Of note, all etiologies share one common feature, which is the proximal tubular injury accompanied with inflammation and immune activation (Basile et al., 2012; Chevalier, 2016; Xu and Han, 2016). Proximal tubular injury can be acutely detrimental to the kidney as well as to the whole organism by impairing key kidney functions, such as reabsorption and secretion, and can lead to long-term problems (e.g., transitioning to CKD and increased risk of CKD and eventual death even after a complete recovery) (Bucaloiu et al., 2012; Jones et al., 2012). Of note, proximal tubules are highly vulnerable to injuries due to the high demand of oxygen consumption, which is required for multiple transport processes, and a relative paucity of endogenous antioxidant defenses (Chevalier, 2016). Thus, proximal tubules are the major target of AKI, and, in line with this, clinically relevant studies demonstrate that molecular targeting of the proximal tubule is sufficient to induce AKI and its transition to CKD (Chevalier, 2016).

For studying AKI, a number of experimental techniques have been developed to directly or indirectly target the kidney, including: (1) surgical approaches – UUO, IRI, CLP (via sepsis), etc.; (2) systemic administration of drugs or toxins inducing nephrotoxicity – injection of cisplatin, glycerol (via rhabdomyolysis), bacterial LPS (via sepsis) etc. (Ramesh and Ranganathan, 2014; Ortiz et al., 2015; Rabb et al., 2016; Bao et al., 2018; Johnson and Zager, 2018); and (3) selective depletion of proximal tubules in genetically modified mice, i.e., injecting mice with DT, which express human DT receptor (DTR) on proximal tubules – *Ggt1*-DTR (Zhang et al., 2012, 2017; Wang et al., 2015), *Ndgr1*-Cre^ERT^:iDTR (Takaori et al., 2016), etc. Importantly, molecular mechanisms of AKI progression may differ depending on the type of insult to the proximal tubule. In effect, methods using (1) septic versus aseptic approaches, (2) systemic versus local, and (3) mild versus severe insults may involve different signaling pathways. This point is well illustrated in studies showing that the nucleotide-binding oligomerization domain, leucine rich repeat and pyrin domain containing 3 (NLRP3) inflammasome pathway is activated in ischemic, but not cisplatin-induced AKI (Kim et al., 2013). As AKI is a major risk factor for CKD progression, CKD is often assessed as a readout for AKI, and experimental AKI models are commonly utilized in the CKD research (detailed information in Tanaka et al., 2014; Chou et al., 2017; Fiorentino et al., 2018). Of note, not all experimental AKI are irreversible and lead to CKD (Chawla et al., 2011; Basile et al., 2012). Overall, it is important to determine the appropriate AKI model depending on the question being asked by giving consideration to the possibility that findings may not be transferable between experimental models. While designing the experiment, we need to consider the pathophysiology of mouse models and the mode of action of tested drugs and specify the model type, functional determination and time course of tissue collection (Ramesh and Ranganathan, 2014). Limitations and pitfalls of animal AKI models as well as the differences between AKI models have been recently described in several reviews. For a comparative overview of the various AKI animal models, please refer to the reviews: Ortiz et al. (2015), Rabb et al. (2016), and Bao et al. (2018).

## Mϕ in AKI

### Infiltrating Mϕ in the Initial Phase of AKI

In experimental AKI models, blood-circulating Ly6C^high^ monocytes are recruited to the inflamed kidney as early as within 1 h (Li and Okusa, 2010; Zhang et al., 2012). The migration of Ly6C^high^ monocytes to the site of inflammation occurs through chemotactic mechanisms (e.g., via CCR2 and CX_3_CR1). Therefore, deletion or blockage of chemotaxis receptors on monocytes is found to be protective against ischemia-induced AKI in mice (Furuichi et al., 2003; Li et al., 2008; Lu et al., 2008; Oh et al., 2008; Yang et al., 2019). Monocyte infiltration occurs in the first 48 h (Lu et al., 2008) and completely ceases before day 3 of AKI. Accordingly, studies showed that the number of Ly6C^high^ monocytes and M1-like Mϕ drastically declines before day 3 of IRI (Lee et al., 2011; Lever et al., 2019) and CX_3_CR1-dependent monocyte migration is not detectable at day 3 of UUO (Peng et al., 2015). Interestingly, the peak of tubular injury [e.g., following IRI (Lee et al., 2011) and glycerol injection (Bussolati et al., 2005)] timely overlaps with the maximum presence of infiltrating monocytes, indicating a close spatial and temporal relationship between the tissue destruction and the accumulation of infiltrating monocytes.

Ly6C^high^ monocytes differentiate into Mϕ, which are primarily skewed toward an M1 phenotype. M1 Mϕ polarization is mediated by pro-inflammatory cytokines [e.g., IFN-γ, IL-6, IL-1β, IL-23, IL-17, C3, C5a, and C5b (Rabb et al., 2016)] and DAMPs [e.g., high mobility group protein B1 (HMGB1), adenosine triphosphate (ATP), uric acid, or hypomethylated DNA (Anders, 2010; McDonald et al., 2010)] released by dying cells or damaged ECM (Anders and Schaefer, 2014). Most recently, soluble epoxide hydrolase was identified as a proximal tubular factor driving M1 polarization of Mϕ in IgA nephropathy (Wang Q. et al., 2018). DAMPs activate various PRRs [e.g., TLR families (Kulkarni et al., 2014; Leaf et al., 2017), NLRP3 and purinergic receptors (Anders and Schaefer, 2014)] on Mϕ and parenchymal cells (Leaf et al., 2017). The importance of DAMPs in inducing innate immune responses is highlighted by findings that the inhibition of PRR signaling suppresses immune responses in AKI (Kim et al., 2013; Leaf et al., 2017). Similar mechanisms are known from acute injuries in other organs, corroborating that DAMPs are central to the immune activation during tissue injury (Egawa et al., 2013; Wang et al., 2014; Groves et al., 2018). Interestingly, TLR4 activation was also shown to induce the expression of IL-22 in Mϕ, which is protective against AKI accelerating kidney regeneration (Kulkarni et al., 2014). M1 polarization of infiltrating Mϕ is additionally supported by parenchymal factors [e.g., Krüppel-like factor 5 (KLF5) expressed by collecting ducts (Fujiu et al., 2011) and suppressor of cytokine signaling 3 (SOCS3) upregulated by proximal tubules in AKI (Susnik et al., 2014)]. Both KLF5 and SOCS3 promote M1 activation of Mϕ and inhibit the expansion of M2 Mϕ in AKI (Fujiu et al., 2011; Susnik et al., 2014). M1-activated Mϕ largely produce pro-inflammatory cytokines and mediators (e.g., IL-1α, IL-6, IL-12, IL-18, TNF-α, nitric oxide), in turn, exacerbating the kidney inflammation (Li and Okusa, 2010).

### M2 Polarization of Infiltrating Mϕ in the Resolution Phase of AKI

**FIGURE 4:**
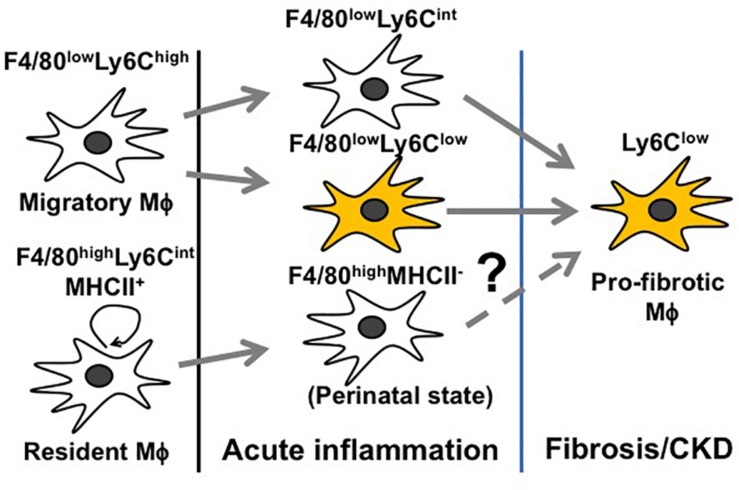
Schematic representation of Mϕ subtypes throughout different stages of AKI.

Inflammation following a transient insult is meant to prepare the tissue for healing. When the inflammation escalates (before day 3 of AKI), Mϕ seek to counteract overwhelming immune activation by skewing toward an immunosuppressive M2 Mϕ to restore tissue homeostasis (Figure 3) (Lee et al., 2011; Baek et al., 2015). However, this only depicts the global view of Mϕ dynamics and does not resolve how individual Mϕ subtypes change during AKI. Since Mϕ are highly plastic and rapidly adapt to the tissue microenvironment, it is difficult to trace the development of individual Mϕ subtypes during AKI. Nevertheless, we are steadily expanding our knowledge base through genetic fate mapping studies and parabiosis experiments. Earlier fate mapping studies revealed that Ly6C^high^ monocytes infiltrating the inflamed kidney give rise to Ly6C^low^ and Ly6C^int^ Mϕ, both phenotypically resembling tissue-resident Mϕ (Lin et al., 2009) (Figure 4). Several studies have shown that monocyte-derived Ly6C^int^ and Ly6C^low^ Mϕ populations display transcriptionally and functionally distinct M2 phenotypes, both implicated in immunosuppression and tissue regeneration. In the later stages of AKI, Ly6C^low^ Mϕ predominate over Ly6C^int^ Mϕ and are found to promote interstitial fibrosis (Lin et al., 2009; Clements et al., 2016; Lever et al., 2019; Yang et al., 2019) (Figure 4). More recent studies revealed that quiescent tissue-resident Mϕ remain in the tissue independently of monocyte-derived Ly6C^low^ Mϕ (Lin et al., 2009; Zhang et al., 2012; Lever et al., 2019) and are reprogramed in AKI toward a developmental state resembling perinatal Mϕ (Schulz et al., 2012; Mass et al., 2016), which are implicated in early kidney development (Lever et al., 2019). These cells display a unique transcriptional profile complying with neither canonical M1 nor M2 nor quiescent Mϕ phenotypes during the first 3 days after IRI. Interestingly, they activate the canonical wingless-type MMTV integration site family (Wnt) signaling by expressing Wnt ligand genes and downstream intracellular signaling mediators, implying that they mediate kidney healing after AKI (Lever et al., 2019). How reprogramed kidney-resident Mϕ further develop in the later stages of AKI and whether they are related to interstitial fibrosis following AKI deserve further investigation (Figure 4).

### Beneficial Effects of M2-Activated Mϕ in AKI

Beneficial effects of M2-activated Mϕ in AKI are supported by many findings: (1) M2 Mϕ clear intraluminal debris (e.g., by apoptosis inhibitor of Mϕ [AIM]-dependent mechanisms) (Arai et al., 2016); (2) secrete tissue-reparative factors, which limit cell cycle arrest (Lin et al., 2010) or apoptosis (Lin et al., 2010; Sola et al., 2011) or which support proliferation in tubular cells (Schmidt et al., 2013) [e.g., Wnt-7b (Lin et al., 2010), lipocalin-2 (Sola et al., 2011), breast regression protein 39 (BRP-39) (Schmidt et al., 2013)]; (3) secrete anti-inflammatory cytokines, which suppress effector T cells or activate regulatory T cells (e.g., IL-10, TGF-β) (Chen et al., 2019); and (4) reduce neutrophil infiltration by downregulating intracellular adhesion molecule-1 (ICAM-1) (Karasawa et al., 2015) and potentially also by sequestering the tissue damage through “cloaking” mechanisms as found in the peritoneal serosa (Uderhardt et al., 2019) etc. As M1 Mϕ are converted into M2 Mϕ in the resolution phase of AKI, a number of studies have focused on identifying stimuli driving Mϕ phenotypic switch during AKI. These stimuli include: (1) paracrine factors released from parenchymal and immune cells; (2) systemic factors, which are released into the blood circulation; and potentially (3) (tissue micro)environmental changes [e.g., apoptotic neutrophils (Filardy et al., 2010; Lee et al., 2011), oxygen (Raggi et al., 2017), and nutrient availability (Geeraerts et al., 2017)].

### Paracrine Factors Released by Proximal Tubules

As mentioned above, proximal tubules are the main locale of the inflammation and potent producers of cytokines in AKI. Therefore, it would not be surprising if they substantially contributed to the phenotypic switch of Mϕ in AKI. In proximal tubule/Mϕ co-culture experiments (proximal tubules and Mϕ physically separated), quiescent proximal tubules are capable of polarizing both non- and M1-activated Mϕ toward an M2 Mϕ phenotype in a paracrine manner, similarly as known from mesenchymal stem cells (Lee et al., 2011; Huen et al., 2015). Unfortunately, the proximal tubular mechanisms of M2 polarization are still largely elusive, and it is also unclear whether the contribution of proximal tubules or proximal tubule-derived factors is indispensable for M2 Mϕ polarization in the resolution phase of AKI. Several studies suggest that proximal tubule-derived Mϕ survival factors (e.g., CSF-1, -2, IL-34) drive M2 Mϕ polarization, but these results remain controversial as discussed in the following paragraph. Proximal tubule-derived factors identified to polarize Mϕ toward an M2 phenotype are Wnt ligands and netrin-1, which are both upregulated during AKI (Reeves et al., 2008; Grenz et al., 2011; Ranganathan et al., 2013; Feng et al., 2018a, 2018b). It has been shown that the blockade of Wnt/β-catenin signaling diminishes M2 Mϕ polarization also reducing interstitial fibrosis in AKI (Feng et al., 2018a, 2018b). Netrin-1 deficiency was found to aggravate AKI, whereas the adoptive transfer of netrin-1-treated Mϕ was protective against AKI (Reeves et al., 2008; Grenz et al., 2011; Ranganathan et al., 2013). Proximal tubules also express both transforming growth factor β (TGF-β) and its receptors at high levels. While TGF-β with its pleiotropic effects acts on various cell types, it is known to polarize Mϕ toward an anti-inflammatory (Wang et al., 2005) and pro-fibrotic phenotype (Braga et al., 2015). However, it is unclear how beneficial the Mϕ-specific effects of TGF-β are on AKI as TGF-β can signal directly to proximal tubules and induce proximal tubular apoptosis (Nath et al., 2011; Gewin et al., 2012). In addition, TGF-β may promote the persistence of fibrotic M2 Mϕ and mediate interstitial fibrosis (Martinez et al., 2009; Lech and Anders, 2013; Chung et al., 2018).

### Controversial Roles of Mϕ Survival Factors in Mϕ Polarization

CSF-1 and -2 are produced and up-regulated by proximal tubules during AKI. Many studies have pinpointed Mϕ survival factors, CSF-1 (Menke et al., 2009; Alikhan et al., 2011; Zhang et al., 2012; Wang et al., 2015) and -2 [also known as granulocyte-Mϕ CSF (GM-CSF)] (Huen et al., 2015), as factors driving the phenotypic switch toward an M2 phenotype, but this feature of Mϕ survival factors is highly controversial. CSF-1 and -2 are commonly used for generating *in vitro* Mϕ from bone marrow or blood monocytes, both being sufficient for Mϕ differentiation and maturation. Since CSF-1 and -2 mature and induce expression of distinct patterns of functional genes after a sufficient culture period, researchers have been incited to determine the polarization potential of CSF-1 and -2 and were led to propose that CSF-1 give rise to a more M2-like and CSF-2 to more M1-like expression patterns in Mϕ *in vitro* (Lacey et al., 2012). Nevertheless, it is important to understand that the translatability of *in vitro* data is limited as CSF-1 and -2 show *in vitro* M2 polarization potential only at high concentrations (Lutz et al., 2000; Hume and MacDonald, 2012; Huen et al., 2015) while being efficient at maintaining Mϕ already at low concentrations (Hamilton et al., 1988; Lutz et al., 2000; Meshkibaf et al., 2014). Whereas a number of studies have claimed that both CSF-1 and -2 drive M2 skewing of Mϕ in mice with AKI (Zhang et al., 2012; Huen et al., 2015; Wang et al., 2015), it was controversially found that IL-34, another ligand for CSF-1R, does not polarize Mϕ in murine AKI (Baek et al., 2015) and lupus model (Wada et al., 2019), indicating that CSF-1R signaling is dispensable in M2 Mϕ polarization. Supportive of this data, other studies have shown that: (1) increased CSF-1 expression in the resolution phase of AKI is not sufficient to prevent Mϕ from M1 polarization when Mϕ are exposed to an M1 stimulus or when they are deprived of an M2 stimulus during AKI (Fujiu et al., 2011; Susnik et al., 2014; Chiba et al., 2016); (2) quiescent and M2 Mϕ in the resolution phase of AKI differ in transcriptional profiles and functions (Lever et al., 2019; Yang et al., 2019); and (3) the sustained blockage of CSF-1R or the constitutive deletion of CSF-1 ameliorates AKI (Lenda et al., 2003; Ma et al., 2009; more discussion in *Assessing M*ϕ *functions by depleting M*ϕ section). Nevertheless, what is consistent throughout all studies (Zhang et al., 2012; Baek et al., 2015; Huen et al., 2015; Wang et al., 2015; Chiba et al., 2016) is that the deficiency in Mϕ survival factors reduces the number of Mϕ (including that of M2 Mϕ predominating in the resolution phase of AKI). It is interesting to note that the deletion of proximal tubule CSF-1 or the blockade of CSF-2 in AKI leads to a reduction in expression of M2 Mϕ-specific genes, which appears modest (<30%; except regarding *Arg1* expression), which may have resulted from the altered ratio of infiltrating and kidney-resident Mϕ, as blood-circulating Mϕ are not affected by the deletion of proximal tubule CSF-1 or the blockade of CSF-2 (Huen et al., 2015; Wang et al., 2015). The observation that clodronate-induced Mϕ depletion increased initial AKI and reduced recovery in the absence of proximal tubule CSF-1 (Wang et al., 2015) provided additional evidence that CSF-1 is not required or sufficient for M2 Mϕ polarization (also commented in Perry and Okusa, 2015). Taken together, CSF-1, -2 and IL-34 are likely not sufficient to polarize Mϕ toward M2 phenotype, but promote the expansion of M2 Mϕ post-AKI (Chiba et al., 2016). It would be interesting for future work to explore whether Mϕ survival factors have only redundant functions and, if not, what the unique, non-overlapping, functions of these Mϕ survival factors are.

### Other Factors Inducing M2 Polarization of Mϕ

Th2 cytokines, IL-4, -10, and -13, are detected in the resolution phase of AKI, but they are not functionally expressed in proximal tubules (Andres-Hernando et al., 2017; Zhang et al., 2017). IL-4 and -13 are produced by Th2 T cells, basophils, mast cells, and granulocytes, whereas IL-10 is produced by regulatory T cells (Liu et al., 2011), B cells as well as Mϕ, induced by prostaglandins, glucocorticoids, apoptotic cells, and G protein-coupled receptor ligands (Zhang et al., 2017). IL-10, which is part of negative feedback response to inflammation and expressed along with pro-inflammatory cytokines, is released into the local tissue and blood circulation and contributes to the suppression of AKI (Deng et al., 2001; Wan et al., 2014; Greenberg et al., 2015; Zhang et al., 2015). Circulating pentraxin-2, also known as serum amyloid P, is found to facilitate the uptake of apoptotic cells and to bind to Fcγ receptors by opsonizing apoptotic cells. This process triggers IL-10 expression and M2 polarization in infiltrating Mϕ (Castano et al., 2009). IL-4 and -13 activate IL-4Rα and its downstream signaling molecule STAT6 and mediate tissue repair and IL-10 immunosuppression (Zhang et al., 2017). IL-4-stimulated Mϕ, not M1-stimulated Mϕ, promote tubular cell proliferation (Lee et al., 2011). Locally synthesized RA, most likely produced by peritubular Mϕ, represses M1 Mϕ and activates RA signaling in the injured tubular epithelium, which, in turn, promotes M2 polarization, thereby reducing Mϕ-dependent injury post-AKI (Chiba et al., 2016). As mentioned above, the proximal tubular mechanisms of M2 Mϕ polarization are only partially understood and deserve more investigation in the future.

### M2 Mϕ in the Progression of CKD

AKI is reversible as long as the cause has been eliminated and the tissue has not been structurally damaged (Chawla et al., 2011; Basile et al., 2012). So far, it is largely unknown which mechanisms determine full recovery versus subsequent CKD after AKI. For a full recovery after AKI, two conditions regarding Mϕ need to be fulfilled: (1) re-transforming and/or removal of pro-fibrotic M2 Mϕ; and (2) the decline in Mϕ numbers to the basal level. Importantly, Mϕ numbers during AKI are supposed to be strictly controlled across all stages, and uncontrolled hyper-proliferation or inadequate removal of M2 Mϕ in the resolution phase of AKI may cause a non-resolving inflammation and chronic pathology as we observe in other disease areas (e.g., in muscle inflammation (Iwata et al., 2012; Baek et al., 2015; Baek et al., 2017). So far, virtually nothing is known about the fate of Mϕ after the tubular repair is complete, and this needs to be investigated in the future (Huen and Cantley, 2017). Since M2 Mϕ are considered to be protective in AKI, there is a growing interest to use M2 Mϕ and Mϕ-modulating agents as therapeutic tools to treat patients with AKI. However, it is to note that M2 Mϕ are considered to be instrumental in the development of pathological fibrosis and the progression of CKD (Duffield, 2010; Anders and Ryu, 2011). Especially, several studies have identified monocyte-derived Ly6C^low^ Mϕ, which predominate over Ly6C^int^ Mϕ in the later stages of AKI, as direct or indirect contributors to interstitial fibrosis (Lin et al., 2009; Anders and Ryu, 2011; Clements et al., 2016; Lever et al., 2019; Yang et al., 2019) and as a hallmark of CKD progression post-AKI. In line with this, CX_3_CL1-CX_3_CR1-mediated survival of Ly6C^low^ Mϕ correlates with interstitial fibrosis in obstructed kidneys (Peng et al., 2015). M2 Mϕ may take an important pro-fibrotic role (1) by promoting the formation of a provisional ECM (containing fibrin, fibrinogen, and fibronectin), which mediates the recruitment of fibrocytes, giving rise to myofibroblasts (= the effector cells in fibrosis, which, in turn, produce large amounts of ECM components); (2) by expressing matrix metalloproteases, some of which serve as essential drivers of fibrosis; and (3) by secreting large amounts of pro-fibrotic factors, which activate and differentiate resident fibroblasts and infiltrating fibrocytes into myofibroblasts [e.g., TGF-β1 and PDGF, vascular endothelial growth factor (VEGF), insulin-like growth factor 1 (IGF1), Galactin-3] (Vernon et al., 2010; Lech and Anders, 2013; Braga et al., 2015; Wynn and Vannella, 2016); and potentially (4) by directly transitioning into myofibroblasts to mediate interstitial fibrosis via a mechanism named “Mϕ-myofibroblast transition (MMT)” (Nikolic-Paterson et al., 2014; Meng et al., 2016; Wang et al., 2016; Wang Y.Y. et al., 2017; Liang et al., 2018; Tang et al., 2018).

## Assessing Mϕ Functions by Depleting Mϕ

As discussed in detail above, Mϕ are highly implicated in AKI and in the progression of CKD, and Mϕ have versatile functions and are like double-edged swords being both tissue-destructive and -suppressive depending on circumstances. To successfully develop Mϕ-based therapeutic approaches for AKI and its outcomes, we need to precisely understand the role of Mϕ and Mϕ subtypes in AKI. To assess Mϕ functions in AKI, a number of studies have addressed how AKI is affected if global Mϕ or individual Mϕ subtypes are removed or reduced throughout different stages of AKI (Figure 1).

Acute kidney injury encompass both an injury phase and a resolution phase (Huen and Cantley, 2017). Mϕ change their functional phenotype throughout different stages of AKI: Mϕ are predominantly skewed toward M1 phenotypes at early stages of inflammation and toward M2 phenotypes in the resolution phase of AKI. As the phenotypic change of Mϕ during AKI has been well characterized and is known to be a time-controlled process, the role of an individual Mϕ subtype can be assessed by depleting the individual Mϕ subtype by deleting the global Mϕ pool at a selected time point (i.e., when the Mϕ subtype predominates). A number of studies respectively pinpointed the role of M1 and M2 Mϕ using various Mϕ depletion strategies during and after the induction of AKI, including: (1) systemic administration of Mϕ-depleting clodronate or (2) neutralizing CSF-1R antibody; and (3) DT injection in mice expressing DTR on Mϕ. We note that in these studies investigators could not limit their depletion strategies to only M1 or M2 Mϕ as the time-specific removal of individual Mϕ subtypes only relates to the change in the relative abundance of the M1/M2 phenotypes at different time points. Of note, there may be a discrepancy between experimental AKI models in disease kinetics and reversibility and also a discrepancy between Mϕ depletion methods. In line with this, a study showed that Mϕ depletion by clodronate at a single dose effectively reduces blood monocytes, but not completely depletes tissue-resident Mϕ and depleted tissue-resident Mϕ are completely replenished within 72 h (Puranik et al., 2018). Other studies suggested that Mϕ depletion by anti-CSF-1R primarily depletes activated resident monocytes, not affecting the numbers of pro-inflammatory monocytes (MacDonald et al., 2010) and the injury (Wynn and Vannella, 2016).

### Impact of Global Mϕ Depletion on AKI and Its Outcomes

To determine the role of global Mϕ, regardless of polarization state, in AKI, studies have been performed using depletion methods based on: (1) repeated administration of Mϕ-depleting agents (clodronate, small molecule CSF-1R inhibitors, neutralizing anti-CSF-1R antibodies, etc.); (2) genetic deletion of Mϕ survival factors (CSF-1, IL-34 or CSF-1R deficiency) (Lenda et al., 2003; Ma et al., 2009; Baek et al., 2015). In general, a (partial) global depletion of Mϕ was revealed to mitigate AKI in UUO and IRI experiments resulting in reduced tubular apoptosis (Lenda et al., 2003; Kitamoto et al., 2009; Ma et al., 2009; Baek et al., 2015) and interstitial fibrosis (Ma et al., 2009; Baek et al., 2015; Liu et al., 2018) (Table 1). In an experimental model of hypertension, the sustained depletion of global Mϕ was shown to attenuate hypertensive renal injury and fibrosis as well as to lower blood pressure (Huang et al., 2018). Much to our surprise, the reduced number of M2 Mϕ in *Il34*^–/–^ mice did not show a delay in the kidney recovery, but prevented kidney fibrosis, being clearly beneficial to the injured kidney (Baek et al., 2015). Remarkably, a UUO experiment showed that the global depletion of Mϕ reduces tubular apoptosis, but does not affect interstitial fibrosis (Ma et al., 2009), but this may be due to the specificity of UUO, where the renal insult is irreversible and the suppression of the injury driving the fibrotic response is more difficult than in other models (Nikolic-Paterson et al., 2014).

**TABLE 1:** Impact of global Mϕ depletion on AKI and its outcomes.

**AKI model**	**Depletion method**	**Outcomes**
**Impact of global Mϕ depletion on AKI and its outcomes: beneficial**		
UUO	Clodronate (before and at day 2 and 4 of UUO)	Reduced tubular apoptosis and fibrosis (Kitamoto et al., 2009)
UUO	Small molecule CSF-1R inhibitor (Fms-I; starting before UUO and 2× daily)	Reduced tubular apoptosis; no change in fibrosis (Ma et al., 2009)
UUO	CSF1 deficiency (knockout)	Reduced tubular apoptosis (Lenda et al., 2003)
Unilateral IRI	IL-34 deficiency (knockout)	Improved kidney function; reduced fibrosis (Baek et al., 2015)
Hypertension (high dose angiotensin II injections)	Clodronate (before and every 3 days till the end of the experiments)	Reduced renal injury and fibrosis; lowered blood pressure (Huang et al., 2018)
UUO	Clodronate (every 2 days starting day 1 before UUO)	Reduced fibrosis (Liu et al., 2018)
**Impact of global Mϕ depletion on AKI and its outcomes: harmful**		
DT-induced depletion of *Ggt1*-expressing proximal tubules	Clodronate or DT-induced depletion of CD11c^+^ cells	Reduced survival (Zhang et al., 2012)
DT-induced depletion of *Ggt1*-expressing proximal tubules or unilateral IRI	Proximal tubule-specific CSF1 deficiency (conditional knockout)	Delayed functional + structural recovery from AKI; increased fibrosis (Wang et al., 2015)

When AKI was induced by cell-specific depletion of proximal tubules in *Ggt1*-DTR mice, global depletion of Mϕ led to opposite results, aggravating AKI. In this specific AKI model, global depletion of Mϕ resulted in reduced survival of mice (Zhang et al., 2012), delayed functional and structural recovery from AKI and increase in interstitial fibrosis (Wang et al., 2015) (Table 1). However, it is important to mention that this AKI model does not involve a prominent Mϕ infiltration as seen in other IRI models (Figure 6 in Zhang et al., 2012), indicating that the initial injury is independent of M1 Mϕ and depletion of global Mϕ mainly targets the resident Mϕ-derived M2 pool.

Overall, these studies indicate that (partial) general depletion of Mϕ is rather beneficial than harmful to the injured kidney, especially in AKI settings where Mϕ infiltration and M1 Mϕ are prominent features (e.g., IRI and UUO models). The significance of Mϕ in tissue repair after AKI is unquestioned as Mϕ are known as extremely potent phagocytes supposed to accelerate the tissue recovery by clearing debris. However, studies showed that the kidney epithelium possess its own mechanisms to self-heal, e.g., by producing autocrine factors, which mediate tubular regeneration [CSF-1 (Menke et al., 2009), TGF-β1 (Gewin et al., 2012) etc.], and Mϕ may not be the only phagocytes in the injured tissue. So far, we do not know whether Mϕ are indispensable in tissue repair after AKI.

### Impact of M1 Mϕ Depletion on AKI and Its Outcomes

To examine the impact of M1 Mϕ on AKI and its outcomes, Mϕ were depleted by injecting clodronate into mice before the induction of AKI by either uni- or bilateral IRI (Day et al., 2005; Jo et al., 2006; Vinuesa et al., 2008; Lee et al., 2011; Ferenbach et al., 2012; Lu et al., 2012) or glycerol injection (Kim et al., 2014) (Table 2). All of these experiments demonstrated that reducing M1 Mϕ prevents immunopathology and improves kidney function in injured kidneys. In one of these studies, the depletion of M1 Mϕ paradoxically showed a reduced tubular regeneration at day 3 of bilateral IRI (Vinuesa et al., 2008); but, this may reflect that tubules were less damaged due to the depletion of M1 Mϕ. Independently, M1 Mϕ removal by immunotoxin (Fet et al., 2012) or neutralizing anti-CSF-1R antibody (Clements et al., 2016) prior to IRI uncovered similar findings including improved kidney function (Fet et al., 2012; Clements et al., 2016) and pathology and reduced oxidative stress (Fet et al., 2012) (Table 2). On the other hand, the reduction of M1 Mϕ by clodronate injection (Lu et al., 2008) and by DT injection in *Cd11b*-DTR mice (± clodronate) did not show any effect on cisplatin- and ischemia-induced AKI, respectively (Ferenbach et al., 2012; Lu et al., 2012) (Table 2). Interestingly, Mϕ depletion by clodronate injection improved AKI in the same IRI study (Ferenbach et al., 2012), indicating that DT-induced depletion of CD11b^+^ cells in *Cd11b*-DTR mice may have affected a larger variety of immune cells including immunosuppressive cell types. Overall, the impact of M1 Mϕ depletion on AKI and its outcomes can be considered as protective in AKI.

**TABLE 2:** Impact of M1 Mϕ depletion on AKI and its outcomes.

**AKI model**	**Depletion method**	**Outcomes**
**Impact of M1 Mϕ depletion on AKI and its outcomes: beneficial**		
Bilateral IRI	Clodronate (before IRI)	Reduced tubular necrosis, apoptosis; reduced inflammation (Day et al., 2005; Jo et al., 2006)
Bilateral IRI	Clodronate (before IRI)	Reduced tubular injury; improved kidney function; but also reduced tubular regeneration (at day 3 of IRI) (Vinuesa et al., 2008)
Unilateral IRI plus contralateral nephrectomy	Clodronate (before IRI)	Reduced tubular injury; improved kidney function (Lee et al., 2011)
Unilateral IRI plus contralateral nephrectomy	Immunotoxin H22(scFv)-ETA (at 6 h of IRI)	Improved histology; less oxidative stress; improved kidney function (Fet et al., 2012)
Unilateral IRI	Clodronate (before IRI)	Improved kidney function; reduced tubular apoptosis (Ferenbach et al., 2012; Lu et al., 2012)
Glycerol injection	Clodronate (before injection)	Reduced tubular apoptosis; reduced inflammation (Kim et al., 2014)
Bilateral IRI	Neutralizing anti-CSF1R antibody (before + at 30 min)	Improved kidney function (Clements et al., 2016)
**Impact of M1 Mϕ depletion on AKI and its outcomes: none**		
Cisplatin injection	Clodronate (before + at day 1)	None (Lu et al., 2008)
Unilateral IRI	Conditional (DT/DTR) ablation of CD11b^+^ cells ± clodronate (before IRI)	None (Ferenbach et al., 2012; Lu et al., 2012)

### Impact of M2 Mϕ Depletion on AKI and Its Outcomes

It may be easy to assume that the expansion of reparative M2 Mϕ in the resolution phase of inflammation would be beneficial to the injured tissue, but, in reality, M2 Mϕ can be both friends and foes in AKI (Braga et al., 2015). Indeed, studies focusing on evaluating the effect of M2 Mϕ depletion in AKI have led to controversial results. On the one hand, depletion of M2 Mϕ (e.g., by clodronate injection or DT-mediated conditional ablation of CD11b^+^ cells) was found to decrease kidney fibrosis (Lin et al., 2009; Kim et al., 2015; Yang et al., 2019), improve the kidney function and reduce the production of inflammatory and pro-fibrotic cytokines in some IRI and UUO experiments (Ko et al., 2008). In addition, M2 Mϕ depletion starting as early as at day 1 after UUO showed an improvement of the immunopathology limiting tissue injury (Yang et al., 2019) (Table 3). Notably, renal fibrosis was found to be reduced in all experiments where M2 Mϕ depletion improves the renal pathology after AKI, indicating that the most undesirable feature of M2 Mϕ in Mϕ-based therapeutic approaches for AKI and CKD is the capability to promote renal fibrosis. Some IRI and septic AKI experiments, on the other hand, led to completely opposite results suggesting that M2 Mϕ depletion is harmful to injured kidneys (Table 3). In these experiments, M2 Mϕ depletion worsened AKI (Li et al., 2018) or delayed the recovery from AKI (Jang et al., 2008; Lee et al., 2011); increased tubular damage and apoptosis (Jang et al., 2008; Menke et al., 2009; Clements et al., 2016) and oxidative stress (Jang et al., 2008); and impaired of kidney function (Menke et al., 2009; Lee et al., 2011; Karasawa et al., 2015; Li et al., 2018). In one study, M2 Mϕ depletion by DT-mediated ablation of CD11b^+^ cells even aggravated kidney fibrosis following IRI, which was contrary to the observations previously mentioned (Menke et al., 2009). In addition, DT-mediated depletion of CD169^+^ cells, which represent tissue-resident M2 Mϕ, markedly worsened the kidney injury and increased the lethality in mice after IRI, which, but, could be rescued by the adoptive transfer of Ly6C^–^ monocytes (Karasawa et al., 2015). Notwithstanding of all above, there was also a study showing that M2 Mϕ depletion (by conditional ablation in CD11b- or CD11c-DTR) does not have any impact on the development of fibrosis after unilateral IRI (Kim et al., 2015) (Table 3). In summary, the impact of M2 Mϕ depletion on AKI and its outcomes was not consistent throughout the experiments, illustrating that M2 Mϕ can be both beneficial and harmful to the injured kidney. These controversial results from AKI studies focusing on elucidating the role of M2 Mϕ corroborate the dual nature of M2 Mϕ. It is interesting to note that M2 Mϕ can to be rather disturbing than useful in the recovery process after AKI depending on the conditions given.

**TABLE 3:** Impact of M2 Mϕ depletion on AKI and its outcomes.

**AKI model**	**Depletion method**	**Outcomes**
**Impact of M2 Mϕ depletion on AKI and its outcomes: beneficial**		
Unilateral IRI plus contralateral nephrectomy	Clodronate (starting on day 3)	Improved kidney function; reduced production of inflammatory and pro-fibrotic cytokines (Ko et al., 2008)
UUO	Conditional (DT/DTR) ablation of CD11b^+^ cells (at day 7–9)	Reduced fibrosis (Lin et al., 2009)
Unilateral IRI	Clodronate (starting on day 3)	Reduced fibrosis (Kim et al., 2015)
Unilateral IRI	Clodronate (starting on day 1)	Improved histology; reduced kidney injury; reduced fibrosis (Yang et al., 2019)
**Impact of M2 Mϕ depletion on AKI and its outcomes: harmful**		
Bilateral IRI	Clodronate (at day 6)	Increased tubular damage; increased oxidative stress; delayed recovery from AKI (?) (Jang et al., 2008)
Unilateral IRI plus CSF-1 injection	Conditional (DT/DTR) ablation of CD11b^+^ cells (at day 1–3)	Increased fibrosis; decreased kidney function; increased apoptosis (Menke et al., 2009)
Unilateral IRI plus contralateral nephrectomy	Clodronate (at day 2 and 3)	Less improvement in glomerular filtration; impaired tubular regeneration (Lee et al., 2011)
Unilateral IRI ± contralateral nephrectomy	Conditional (DT/DTR) ablation of CD169^+^ cells (24–36 h before IRI)	Lethality, failed kidney function, increased inflammation (Karasawa et al., 2015)
Bilateral IRI	Neutralizing anti-CSF1R antibody (at day 1–3)	Increased apoptosis (Clements et al., 2016)
CLP	Clodronate	Worsening AKI; decreased kidney function (Li et al., 2018)
**Impact of M2 Mϕ depletion on AKI and its outcomes: none**		
Unilateral IRI	Conditional (DT/DTR) ablation of CD11b^+^ or CD11c^+^ cells (starting on day 3)	No change in fibrosis (Kim et al., 2015)

## Mϕ-Based Therapeutic Strategies

Mϕ are instrumental in maintaining immune homeostasis and mediating inflammation. Therefore, modulation of Mϕ functions is widely considered as a promising approach for various kidney diseases. Different Mϕ-based strategies have been suggested for the treatment of AKI, including: (1) adoptive transfer of *ex vivo* Mϕ that are M2-activated via (a) treatment with M2 stimuli (Wang et al., 2007; Cao et al., 2010; Ranganathan et al., 2013; Geng et al., 2014) or (b) genetic manipulation (Wilson et al., 2002; Ferenbach et al., 2010; Jung et al., 2012, 2016); (2) adoptive transfer of immunomodulatory cells (such as bone marrow-derived mesenchymal stem cells, umbilical cord-derived stromal cells (Li et al., 2013; Geng et al., 2014; Rota et al., 2018) and type 2 innate lymphoid cells (Huang et al., 2015; Cao et al., 2018); (3) systemic administration of M2-polarizing agents (Cao et al., 2011; Chen et al., 2017; Wang Q. et al., 2017; Wang S. et al., 2017; Barrera-Chimal et al., 2018) [for more detailed information on this topic, please refer to the comprehensive review (Chen et al., 2019)]. Of note, most of these proposed strategies are based on the modulation of Mϕ functions favoring M2 anti-inflammatory state. In such a strategy, the risk of triggering renal fibrosis with M2 Mϕ can be a critical issue (Braga et al., 2015). Thus, studies have also focused on developing genetic modification of *ex vivo* Mϕ to suppress the development of kidney fibrosis. It has been found that the adoptive transfer of Mϕ overexpressing neutrophil gelatinase-associated lipocalin-2 (NGAL) (Guiteras et al., 2017) or lacking legumain (Wang D. et al., 2018) can attenuate renal interstitial fibrosis.

Interestingly, the reduction in the number of global and M2 Mϕ can be beneficial to the injured kidney and a promising approach to treatment of AKI. Actually, Mϕ-depleting clodronate and anti-CSF-1R neutralizing antibodies are used in different clinical areas (Frediani and Bertoldi, 2015; Peyraud et al., 2017; Frediani et al., 2018; Goldvaser and Amir, 2019). A potential target for depleting Mϕ is CSF-1R signaling. *Csf1r*^–/–^ mice and mice deficient in functional CSF-1 (*Csf1*^op/op^ mice) completely lack Mϕ, but also exhibit other severe non-Mϕ-related physiological abnormalities (Wei et al., 2010), illustrating that spatiotemporal expression of CSF-1 is crucial to many important biological processes. It has been found that genetic deletion of IL-34 partially removes Mϕ in injured kidneys and is beneficial in AKI (Baek et al., 2015) as well as in lupus nephritis (Wada et al., 2019). As *Il34*^–/–^ mice show no gross phenotype in steady state (Greter et al., 2012; Wang et al., 2012), targeting of IL-34 appears to be more tolerable than that of CSF-1R or CSF-1. IL-34 may be useful for the partial removal of global Mϕ throughout all stages of AKI or for reducing M2 Mϕ in the later stages of AKI.

## Conclusion and Outlook

This review has provided insights into the net effect of versatile Mϕ functions in AKI by Mϕ removal studies (Figure 1). Interestingly, several studies suggest that the (partial) depletion of global Mϕ in AKI can be beneficial to the injury kidney. In addition, this review has assessed the current literature on the impact of the depletion of individual Mϕ subtypes on AKI and its outcomes and found that M1 Mϕ depletion has been shown to be generally protective against AKI, whereas M2 Mϕ depletion has led to controversial results.

How can we translate findings from animal AKI models into clinical practice? M1 Mϕ instantly enter the tissue within an hour after AKI and phenotypically switch to M2 Mϕ within a couple of days. In most AKI cases, the onset cannot be predicted (e.g., unless patients are scheduled for a kidney transplant or other relevant surgery) and precedes the diagnose. Thus, therapeutic intervention via targeting of M1 Mϕ can be challenging. As M2 Mϕ can resolve inflammation, there is a growing interest to use M2 Mϕ and Mϕ-modulating agents as therapeutic tools to treat patients with AKI (Chen et al., 2019); however, we may not underestimate that M2 Mϕ can contribute to interstitial fibrosis and facilitate the AKI-to-CKD transition. Overall, M2 Mϕ act as double-edged swords being both beneficial and harmful to the inflamed kidney tissue (Braga et al., 2015), and the dual nature of M2 Mϕ is well recapitulated in the results from M2 Mϕ depletion studies. As uncontrolled hyper-proliferation or inadequate removal of Mϕ in the resolution phase of inflammation can cause chronic inflammation and eventual organ failure, we need to simultaneously consider two avenues, when developing therapeutic approaches targeting Mϕ, including: (1) modulation of Mϕ activation and functions and (2) removal of excess Mϕ. Previous studies investigating the role of Mϕ in AKI mostly focused on the mechanism of Mϕ survival, proliferation and polarization, we do not understand by which mechanisms Mϕ disappear in the resolution phase of inflammation. Future studies need to investigate the fate of individual Mϕ subtypes after the tissue repair is completed.

## Author Contributions

J-HB conceptualized and wrote the manuscript.

## Conflict of Interest Statement

J-HB is an employee of Biogen.

## Abbreviations

AKI: acute kidney injury
CKD: chronic kidney disease
CLP: cecal ligation and puncture
CSF: colony-stimulating factor
DAMPs: damage-associated molecular patterns
DT: diphtheria toxin
(i)DTR: (inducible) diphtheria toxin receptor
ECM: extracellular matrix
IRI: ischemia-reperfusion injury/surgery
LPS: lipopolysaccharide
Mϕ: macrophage(s)
PAMPs: pathogen-associated molecular patterns
PDGF: platelet-derived growth factor(s)
PRR: pathogen recognition receptor(s)
RA: retinoic acid
Th: T helper type
TLR: toll-like receptor
UUO: unilateral ureteral obstruction.
